# Mechanistic elucidation of ferroptosis and ferritinophagy: implications for advancing our understanding of arthritis

**DOI:** 10.3389/fphys.2024.1290234

**Published:** 2024-07-03

**Authors:** Caopei Guo, Jiaze Peng, Piaotao Cheng, Chengbing Yang, Shouhang Gong, Lin Zhang, Tao Zhang, Jiachen Peng

**Affiliations:** ^1^ Department of Orthopedics, Affiliated Hospital of Zunyi Medical University, Zunyi, China; ^2^ Joint Orthopaedic Research Center of Zunyi Medical University, University of Rochester Medical Center, Zunyi, China; ^3^ Key Laboratory of Cell Engineering of Guizhou Province, Affiliated Hospital of Zunyi Medical University, Zunyi, China; ^4^ Department of Burn and Plastic Surgery, Affiliated Hospital of Zunyi Medical University, Collaborative Innovation Center of Tissue Damage Repair and Regeneration Medicine, Zunyi, China

**Keywords:** ferroptosis, iron accumulation, lipid peroxidation, ROS, glutathione, GPx4, ferritinophagy, arthritis

## Abstract

In recent years, the emerging phenomenon of ferroptosis has garnered significant attention as a distinctive mode of programmed cell death. Distinguished by its reliance on iron and dependence on reactive oxygen species (ROS), ferroptosis has emerged as a subject of extensive investigation. Mechanistically, this intricate process involves perturbations in iron homeostasis, dampening of system Xc-activity, morphological dynamics within mitochondria, and the onset of lipid peroxidation. Additionally, the concomitant phenomenon of ferritinophagy, the autophagic degradation of ferritin, assumes a pivotal role by facilitating the liberation of iron ions from ferritin, thereby advancing the progression of ferroptosis. This discussion thoroughly examines the detailed cell structures and basic processes behind ferroptosis and ferritinophagy. Moreover, it scrutinizes the intricate web of regulators that orchestrate these processes and examines their intricate interplay within the context of joint disorders. Against the backdrop of an annual increase in cases of osteoarthritis, rheumatoid arthritis, and gout, these narrative sheds light on the intriguing crossroads of pathophysiology by dissecting the intricate interrelationships between joint diseases, ferroptosis, and ferritinophagy. The newfound insights contribute fresh perspectives and promising therapeutic avenues, potentially revolutionizing the landscape of joint disease management.

## 1 Introduction

The phenomenon of cell death stands as a pervasive occurrence within the biological domain, constituting an irrefutable and pivotal facet of natural processes. Cell demise manifests through a spectrum of mechanisms, encompassing regulated cell death (RCD), including apoptosis, pyroptosis, autophagy-dependent cell death, ferroptosis, and immunogenic cell death, in conjunction with accidental cell death (ACD) (Ni et al., 2022; Galluzzi et al., 2018). In recent years, the profound implications of programmed cell death pathways have arisen, emerging as indispensable determinants orchestrating physiological and pathological dynamics. Notably, among these intricate pathways, ferroptosis has emerged as a distinctive mode of cell death contingent on iron and reliant on ROS, a conceptualization originally articulated by Dixon and collaborators in 2012. Importantly, ferroptosis shows distinctive morphological and biochemical attributes. This demarcates it from similar cell death modalities such as apoptosis, necrosis, and autophagy (Dixon et al., 2012) (Table 1). Morphologically, ferroptotic cells assume a characteristic rounded configuration akin to necrotic cells, although bereft of cytoplasmic or organelle engorgement and plasma membrane disruption. Nuclear integrity is upheld, manifesting as preserved structure and size devoid of hallmarks such as chromatin margination, condensation, or apoptotic body formation characteristic of apoptosis (Ke et al., 2016). Notably absent are hallmarks of other modalities, such as the double-membrane enclosed autophagosomes observed in autophagy or the membrane bubbling and integrity compromise seen in pyroptosis (Liang et al., 2019; Li X. et al., 2022). A defining morphological trait of ferroptosis resides in mitochondrial dynamics, encompassing shrinkage, attenuated abundance, augmented membrane density, and the diminishment or obfuscation of cristae (Yu et al., 2017; Han et al., 2020). Biochemically, ferroptosis is distinguished by intracellular iron accumulation, culminating in glutathione (GSH) exhaustion, diminished or impaired activity of phospholipid hydroperoxide glutathione peroxidase 4 (GPX4), a central modulator of ferroptosis, and the accumulation of ROS (Yang et al., 2014). The ensuing cascade engenders lipid peroxidation, which engenders the production of reactive oxygen species, thereby perturbing the selective permeability of the cellular membrane (Stockwell et al., 2017). Importantly, the refractory nature of ferroptosis to inhibitors of apoptosis and autophagy is evident, yet its susceptibility to reversal by iron chelators like deferoxamine, lipophilic antioxidants, lipid peroxidation inhibitors, and polyunsaturated fatty acids remains intriguing (Lei et al., 2019).

**TABLE 1:** Morphological features of ferroptosis, apoptosis, necrosis, autophagy, and pyroptosis.

Classification	Morphological features	References
**Ferroptosis**	1.Mitochondrial alterations: These include mitochondrial volume reduction, mitochondrial cristae disappearance, and increased mitochondrial membrane density	Yagoda et al. (2007), Dixon et al. (2012), Galluzzi et al. (2018), Yu et al. (2021)
	2.Cell volume reduction: The cell undergoes a progressive reduction in volume	
	3.Rupture of the mitochondrial outer membrane: Rupture of the mitochondrial outer membrane can be observed, but the nuclear envelope usually remains intact	
	4.Absence of chromatin condensation or DNA fragmentation	
	5.Lipid peroxidation: lipids in the cell membrane and organellar membranes can be damaged	
**Apoptosis**	1. Cell shrinkage: The cell reduces in size and becomes more rounded	Saraste and Pulkki (2000), Doonan and Cotter (2008), Galluzzi et al. (2018), Bertheloot et al. (2021), Yang et al. (2021b)
	2. Chromatin condensation: Chromatin inside the nucleus becomes condensed and aggregates towards the nuclear envelope	
	3. Formation of apoptotic bodies: The cell’s membrane blebs and forms multiple membrane-bound bodies that are rapidly engulfed by surrounding cells	
	4. Membrane changes: Redistribution of phospholipid molecules in the cell membrane, like the externalization of phosphatidylserine	
	5. DNA fragmentation: DNA within the cell is cleaved into specific-sized fragments	
	6. Absence of inflammation	
**Necrosis**	1. Cellular swelling and membrane rupture: The integrity of the cell membrane is compromised, leading to a release of cell contents into the extracellular environment	Galluzzi et al. (2018), Zhang et al. (2019c), Tonnus et al. (2019), Bertheloot et al. (2021)
	2. Nuclear changes: This involves chromatin condensation (pyknosis), fragmentation (karyorrhexis), and dissolution (karyolysis)	
	3. Cytoplasmic clumping and vacuolation: Organelles and structures within the cell may degrade and appear cloudy, with vacuoles potentially forming in the cytoplasm	
	4. Inflammatory response: Due to the release of cellular contents, there is often an inflammatory response in the surrounding tissue	
	5. Absence of apoptotic body formation	
**Autophagy**	1. Formation of autophagosomes: In the early stages of autophagy, double-membraned vesicular structures known as autophagosomes appear in the cytoplasm. These vesicular structures surround and isolate proteins and organelles within the cell	Mizushima and Komatsu (2011), Parzych and Klionsky (2014), Galluzzi et al. (2018)
	2. Fusion with lysosomes: The autophagosomes fuse with lysosomes to form autolysosomes. Lysosomes contain enzymes capable of degrading the sequestered cellular contents	
	3. Degradation of contents: Within the autolysosome, cellular components are effectively broken down into their basic constituents, such as amino acids and fatty acids, which can be reused by the cell	
	4. Visualization of membranous structures: Under electron microscopy, a variety of membranous structures associated with autophagy, such as early autophagic vacuoles, mature autophagosomes, and autolysosomes, can be distinctly observed	
**Pyroptosis**	1. Membrane blebbing: The cell membrane begins to form small, bubble-like protrusions, known as blebs, without an increase in cell volume	Vande Walle and Lamkanfi (2016), Galluzzi et al. (2018), Bertheloot et al. (2021)
	2.Pore formation in the membrane: Specific inflammatory proteins form pores on the cell membrane	
	3. Release of cellular contents: Due to the pores in the cell membrane, cellular contents, including inflammatory cytokines, are released into the extracellular space	
	4. DNA fragmentation: The DNA within the cell nucleus may undergo fragmentation, but this pattern of fragmentation is different from that in apoptosis	
	5. Chromatin condensation: This condensation is not as pronounced as in apoptosis	

## 2 The mechanism of ferroptosis

Central to the execution of ferroptosis is the accumulation of intracellular iron, orchestrating a cascade of events entailing ROS generation and consequential lipid peroxidation. The complex interplay culminating in ferroptosis can be conceptualized as an orchestrated interplay between oxidative stress and antioxidant defense systems, engendering an intricate imbalance (Kuang et al., 2020). In cases marked by dysregulated cellular iron homeostasis, a resultant decline in intracellular GSH levels coupled with the attenuated activity of GPX4 contributes to an augmented generation of ROS. The ensuing oxidative milieu precipitates interaction between ROS and polyunsaturated fatty acids on the cell membrane, prompting the onset of lipid peroxidation. The threshold where ROS generation surpasses cellular antioxidant capacity culminates in cellular architecture erosion, underscoring the incipient demise (Qin Y. et al., 2021). Regulatory mechanisms underpinning ferroptosis prominently entail disturbances in iron metabolism, concomitant attenuation of the system Xc--GSH-GPX4 axis, escalated ROS levels, and rampant lipid peroxidation. Within the intricate milieu of the cell, discernible actors such as mitochondria, lysosomes, and peroxisomes engage in harmonized choreography, meticulously modulating the generation of ROS and orchestrating the propagation of lipid oxidation. Profoundly, the manifestation and extent of ferroptosis hinge on the intricate and nuanced cross-signaling orchestrated by these cellular envoys (Feng and Stockwell, 2018).

### 2.1 Iron metabolism

**FIGURE 1:**
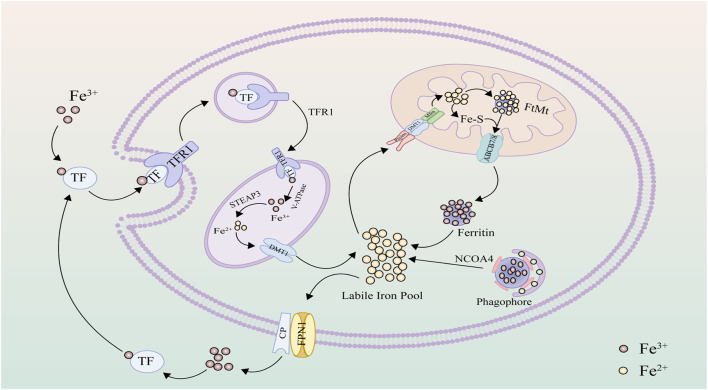
Iron metabolism: Upon engagement with transferrin (TF), ferric iron (Fe3+) is targeted and internalized by the specific interfacing of the transferrin receptor 1 (TFR1). Within cellular confines, the iron undergoes reduction to its ferrous form (Fe2+) under the mediation of the six-transmembrane epithelial antigen of the prostate 3 (STEAP3). Following this, the divalent metal transporter 1 (DMT1) facilitates its translocation into the cytoplasm, where it subsequently resides in the labile iron pool. For mitochondrial uptake, iron traverses through specific pore proteins and DMT1 to reach the mitochondrial intermembrane space and is then incorporated into the mitochondrial matrix via Mfrn. Within the mitochondria, the metal may be compartmentalized in FtMt or, alternatively, channeled out via specialized iron export proteins. In a subsequent event, iron-laden ferritin complexes with the NCOA4 protein, forming autophagosomes. Lysosomal fusion triggers the enzymatic breakdown of these complexes, liberating encapsulated iron ions. In situations of iron abundance, the cell, aided by ceruloplasmin (CP), expels excess via the ferroportin (FPN1).

Iron, an essential nutritional element, plays a pivotal role in biological activities, with replenishment primarily reliant on dietary intake to compensate for internal losses. Within the body, iron predominantly finds storage in hemoglobin within red blood cells (Nemeth and Ganz, 2023). Iron in food is primarily absorbed by duodenal epithelial cells in its Fe3+ form. In the bloodstream, iron forms complexes with transferrin (TF) and is recognized by transferrin receptor 1 (TFR1) on the cell membrane, facilitating endocytosis to form endosomes that enter the cell (Gammella et al., 2017). Subsequently, the vesicular H + -ATPase (V-ATPase) within endosomes acidifies the intravesicular environment, altering the conformation of the TF-TFR1 protein complex to facilitate the release of Fe3+ (Sobh et al., 2020). Inside the cell, Fe3+ is reduced to Fe2+ by the six-transmembrane epithelial antigen of the prostate 3 (STEAP3), and subsequently, divalent metal transporter 1 (DMT1) mediates the translocation of Fe2+ from endosomes to the cytoplasm (Liu P. et al., 2022). A minor fraction of reduced Fe2+ is sequestered within the labile iron pool (LIP) as an intermediary for iron input, storage, and utilization (Zeidan et al., 2021). The metabolically active LIP can transport iron to mitochondria via mitoferrin (Mfrn) (Muckenthaler et al., 2017). The majority of iron exists as ferric iron (Fe3+) stored within ferritin (FTN), predominantly in cytoplasmic ferritin heavy chain (FTH) and mitochondrial ferritin (FtMt) (Fuhrmann and Brüne, 2022). Ferritin forms spherical aggregates composed of ferritin heavy and light chains, acting as a protective entity against oxidative stress due to excessive free iron (Fuhrmann et al., 2020). Surplus iron, with the aid of ferroxidase ceruloplasmin (Cp), undergoes oxidation from ferrous (Fe2+) to ferric (Fe3+) form, facilitated by iron exporter ferroportin 1 (FPN1) to exit the cell (Sukhbaatar and Weichhart, 2018). Ferroportin is the sole known iron export protein, pivotal in iron transport between duodenal epithelial cells, hepatocytes, Kupffer cells, and splenic red pulp macrophages (Donovan et al., 2005; Drakesmith et al., 2015). Following entry into circulation, iron continues to bind transferrin in the plasma, facilitating its transport to various iron-deficient tissues and organs (Tian et al., 2022). Beyond participating in enzyme-catalyzed and non-enzymatic lipid peroxidation reactions (Kajarabille and Latunde-Dada, 2019), iron can also catalyze the decomposition of H2O2 into hydroxyl radicals through the Fenton reaction, contributing to intracellular reactive oxygen species accumulation (Wardman and Candeias, 1996; Liu M. et al., 2022). In cases of abnormal cellular iron metabolism, the excess accumulation of iron prompts intracellular accumulation of reactive oxygen species and lipid peroxidation, resulting in the degradation of cellular constituents such as lipids, DNA, and proteins (Koleini et al., 2021) (Figure 1).

*In vitro* and *in vivo* experiments have indicated that iron overload exacerbates the generation of ROS and mitochondrial damage in MC3T3-E1 cells, inhibits osteogenic capacity, and leads to the development of osteoporosis in rats (Ren et al., 2023). This osteoporosis may be attributed to the intracellular accumulation of iron, which induces G1 cell cycle arrest in osteoblasts, accompanied by downregulation of Cyclin D1, Cyclin D3, CDK2, CDK6, and CDK6 expression, thereby suppressing osteoblast proliferation (Cen et al., 2018). Furthermore, iron overload also disrupts the cell cycle of bone marrow mesenchymal stem cells and inhibits their population expansion (Yuan et al., 2019).

### 2.2 Mitochondrial metabolism

The energy metabolism process occurring within mitochondria holds pivotal importance for the survival of eukaryotic cells. Furthermore, mitochondria are intricately involved in the regulatory orchestration of trace elements (Cheng R. et al., 2022). Notably, alterations in mitochondrial morphology constitute a hallmark feature in cells undergoing ferroptosis. Conspicuously, these alterations are accompanied by distinctive phenomena such as mitochondrial shrinkage, diminished mitochondrial abundance, heightened membrane density, and decreased cristae formation (Dixon et al., 2012; Yu et al., 2017; Han et al., 2020).

Iron plays a central role within mitochondria, where it is notably involved in pivotal processes such as hemoglobin synthesis, the formation of Fe-S clusters, and their storage within FtMt (Lane et al., 2015). Intriguingly, the journey of iron involves traversing through porins and DMT1 to access the intermembrane space. Subsequently, the mitochondrial iron transporter facilitates its transport into the mitochondrial matrix. Upon entering the mitochondria, iron helps form vital iron-sulfur clusters and generates energy in the electron transport chain (ETC). ETC., is compact and efficient. It is responsible for transferring electrons to oxygen via nicotinamide adenine dinucleotide (NADH) and flavin adenine dinucleotide (FADH2) and releasing energy (Raimondi et al., 2020). However, excess iron is not only confined to specific mitochondrial proteins; it can also be exported by specialized proteins like ABCB7 and ABCB8 (Pearson et al., 2020). ABCB7, located on the mitochondrial membrane, transports iron-sulfur clusters to the cytoplasm, regulating iron metabolism and ferroptosis (Lehrke et al., 2021). Meanwhile, ABCB8, found on the inner mitochondrial membrane, maintains cellular iron balance and protects the heart, potentially affecting ferroptosis, with more research needed to define its role (Ichikawa et al., 2014). The repercussions of mitochondrial iron overload include an elevation in free iron content, prompting the Fenton reaction to generate a surplus of reactive oxygen species, thereby intensifying oxidative damage.

Mitochondria, in addition to their crucial role as the epicenter of cellular energy metabolism, also function as the pivotal regulatory center orchestrating cellular apoptosis. Recent investigations underscore the pivotal role of mitochondria as the principal cellular organelles contributing to the generation of ROS. Particularly within the process of oxidative phosphorylation, the electron transport chain engrosses the major portion of oxygen consumption along the inner mitochondrial membrane. A noteworthy occurrence involves the unintended leakage of electrons from, ETC., complex III, leading to the liberation of superoxide (O2 •−) into the confines of the mitochondrial matrix (Homma et al., 2021). Consequent to this, the enzyme superoxide dismutase (SOD) catalyzes the conversion of O2 •− into hydrogen peroxide (H2O2), and the interaction between H2O2 and Fe2+ triggers the formation of hydroxyl radicals (• OH) (Murphy, 2009). This cascade of events subsequently fosters the oxidation of PUFAs, resulting in the production of PUFA hydroperoxides (PUFA-OOH) (Zheng and Conrad, 2020). This trajectory of events ultimately instigates lipid peroxidation, thereby engendering structural and functional impairments within the mitochondria.

### 2.3 System Xc-—GSH/GPX4 axis

GSH is a pivotal antioxidant within the ferroptosis pathway, essential for maintaining redox equilibrium. Intracellular glutathione originates from extracellular cysteine. System Xc-constitutes a sodium-independent cysteine/glutamate antiporter system, composed of the solute carrier family 7 member 11 (SLC7A11) and the solute carrier family 3 member 2 (SLC3A2). This dyad orchestrates transmembrane transport of extracellular cysteine and intracellular glutamate in a 1:1 ratio through an ATP-dependent mechanism (Zhou X. et al., 2022). Intracellular cysteine is reduced to cystine and subsequently transformed into reduced glutathione through the concerted actions of glutamate cysteine ligase (GCL) and glutathione synthetase (GS) (Bayır et al., 2020). GPX4 stands as a pivotal regulator within ferroptosis, assuming significance as a member of the selenoprotein family. GPX4 serves as the linchpin in reducing polyunsaturated fatty acids (PUFAs) to nontoxic lipid alcohols. Concurrently, it converts reduced glutathione into oxidized glutathione (GSSG), serving as a co-factor to alleviate oxidative stress-induced damage (Yang et al., 2016; Chen et al., 2021). Consequently, suppression of system Xc-activity significantly diminishes intracellular GSH synthesis, thereby impinging on subsequent GPX4 efficacy in peroxide clearance (Wang et al., 2020). In the event of GPX4 inactivation or degradation, the accumulation of toxic peroxides within cells burgeons, leading to the generation of membrane lipid oxygen radicals, culminating in the instantiation of ferroptosis (Ursini and Maiorino, 2020).

### 2.4 ROS and lipid peroxidation

A pivotal phase in the ferroptosis cascade involves the peroxidation of lipids, leading to the disruption of cellular membranes. Within normal cellular contexts, an intricate equilibrium between lipid oxidation and reduction is meticulously maintained. However, ferroptosis starkly highlights a pronounced escalation in lipid oxidation (Yang et al., 2022). Significantly, lipids serve as vital building blocks within cell membranes, not only fortifying cellular structure but also orchestrating essential regulatory functions (Rochette et al., 2022). Among these lipid constituents, polyunsaturated fatty acids (PUFAs), characterized by bis-allylic carbons, exhibit a remarkable susceptibility to lipid peroxidation reactions (Kagan et al., 2017). The intricate process entails the enzymatic esterification of PUFAs by long-chain acyl-CoA synthetase 4 (ACSL4), giving rise to fatty acyl-CoA esters (PUFA-CoA). Subsequently, these esters undergo a transformative modification catalyzed by lysophosphatidylcholine acyltransferase 3 (LPCAT3), culminating in the formation of PUFA-phosphatidylethanolamines (PUFA-PE), which then seamlessly integrate into the intricate architecture of cellular phospholipid bilayers (Lee et al., 2021). It is within this context that PUFA-PE becomes susceptible to an additional oxidation step catalyzed by lipoxygenases (LOX), ultimately generating phosphatidylethanolamine hydroperoxides (PE-PUFA-OOH) (Shah et al., 2018). The activation of ferroptosis ensues when the levels of PE-PUFA-OOH surpass cellular tolerance thresholds. Notably, byproducts arising from the breakdown of lipid peroxides, such as 4-hydroxynonenal (4-HNE) and malondialdehyde (MDA), engage in consequential interactions with nucleic acids and proteins, intensifying the magnitude of cellular damage experienced (Ayala et al., 2014; Feng and Stockwell, 2018).

**FIGURE 2:**
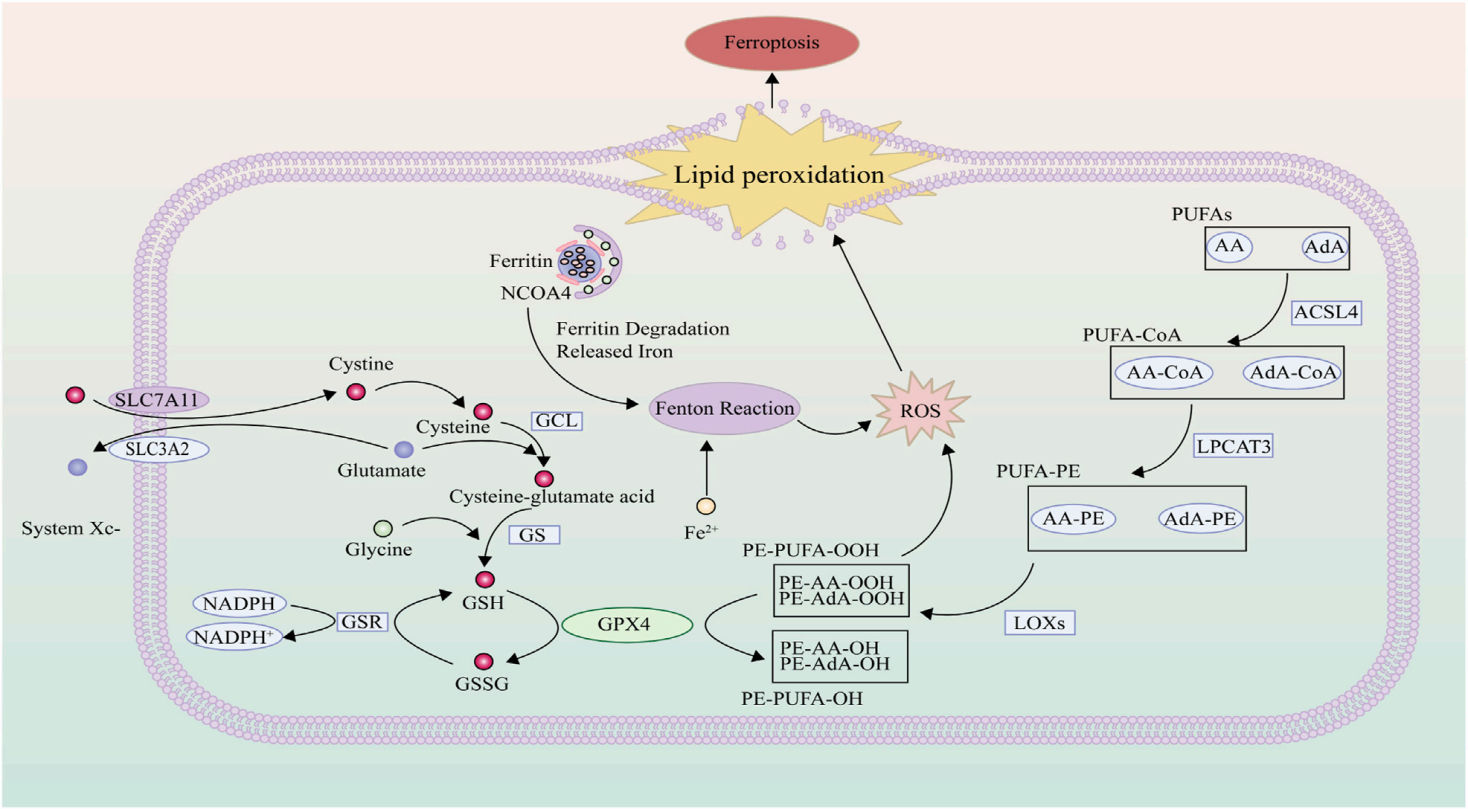
ROS and Lipid Peroxidation: Polyunsaturated fatty acids residing on the cellular membrane undergo a transformation, catalyzed by the concerted action of ACSL4, LPCAT3, and LOX, yielding lipid hydroperoxides. Once these hydroperoxide concentrations eclipse the cellular management threshold, it precipitates the activation of ferroptosis. Intracellularly, cystine is incorporated via the system Xc-, with enzymes such as GCL and GS augmenting its reduction to the bioactive reduced glutathione. In a crucial process mediated by GPX4, the oxidative lipid hydroperoxides are transmuted into benign lipid alcohols, thereby attenuating lipid peroxidation events. However, in scenarios marked by cellular iron superabundance, the Fenton reaction becomes prominently engaged, spawning hydroxyl radicals. This amplifies the proliferation of reactive oxygen species, instigating heightened lipid peroxidation.

Within the electron transport chain, mitochondria generate a substantial amount of ROS even during normal energy metabolism, a role of significant import in cellular signal transduction and tissue homeostasis (Latunde-Dada, 2017). ROS emerges as singlet electron intermediates during the process of oxygen reduction to water, encompassing both free radical and non-radical intermediates (Rochette et al., 2022). These comprise primarily superoxide (O2•−), hydrogen peroxide (H2O2), and hydroxyl radicals (HO•) (Xie et al., 2022). Under ordinary circumstances, the levels of ROS are held at physiological levels through an array of antioxidant enzymes. However, when the pace of ROS production and clearance becomes imbalanced, cellular redox equilibrium falters, allowing ROS to amass progressively, thereby inducing lipid peroxidation through oxidative stress (Cheng G. et al., 2022). Simultaneously, lipid peroxidation may further propagate ROS-induced damage to DNA, lipids, and proteins (Tripathi et al., 2022). Moreover, iron ions, activated through redox processes, can catalyze the decomposition of H2O2 via the Fenton reaction, yielding highly toxic superoxide radicals that directly trigger ferroptosis (Li J. et al., 2022) (Figure 2).

## 3 The connection between ferritinophagy and ferroptosis

Ferritinophagy refers to the process of autophagic degradation of FTN. Ferritin consists of 24 subunits, comprising ferritin heavy chain (FTH1) and ferritin light chain (FTL1). It chelates oxidative-reductive active iron and each ferritin molecule can store up to 4500 iron atoms, playing a pivotal role in cellular antioxidation (Fang et al., 2021). Nuclear receptor coactivator 4 (NCOA4) stands as a key factor in ferritinophagy, facilitating the binding of ferritin and subsequently subjecting it to autophagic degradation (Rochette et al., 2022). NCOA4-dependent ferritinophagy involves the release of iron from ferritin, thereby promoting ferroptosis. Serving as a selective autophagy receptor, NCOA4 interacts with arginine residues within the C-terminal domain of FTH1 (Mancias et al., 2015). This interaction directs cellular ferritin to the autolysosome, leading to selective ferritin chelation and degradation. The liberated iron is then released into the LIP of the cytoplasm, enhancing the bioavailability of Fe2+. Under physiological conditions, ferritinophagy helps maintain intracellular iron levels and contributes to sustaining normal mitochondrial function. However, under conditions that either inhibit or promote ferritinophagy, cellular LIP levels correspondingly decrease or increase (Fujimaki et al., 2019). Bafilomycin A1 (BafA1), an inhibitor of vacuolar-type H + -ATPase (V-ATPase) within the lysosome, effectively restrains the increase in LIP levels induced by erastin-induced ferroptosis. Similarly, LIP levels are significantly reduced in autophagy-deficient cells (Gao et al., 2016). An elevation in Fe2+ levels also invoke a negative feedback loop regulating NCOA4 abundance. When cellular iron content is ample, HERC2 (E3 ubiquitin ligase) specifically recognizes and binds to the carboxy-terminal end of NCOA4 via the CUL7 homology region. This leads to ubiquitination of NCOA4 and inhibition of ferritinophagy, curtailing iron release to balance intracellular iron levels. Experimental studies have shown that HERC2 preferentially recognizes NCOA4 containing Fe-S clusters, leading to degradation of NCOA4 and subsequent accumulation of ferritin (Kuno and Iwai, 2023). Conversely, in low iron environments, the interaction between NCOA4 and HERC2 is suppressed, thereby facilitating the release of free iron through the promotion of ferritinophagy to meet cellular iron demands. While NCOA4 binds to HERC2 in an iron-dependent manner, it can mobilize iron in ferritin for the synthesis of heme. When the NCOA4 gene is knocked down, the accumulation of ferritin makes iron unavailable for subsequent synthesis of heme. (Mancias et al., 2015). The intricate relationship between iron excess and ferroptosis becomes evident, suggesting a close connection between NCOA4-mediated ferritinophagy and ferroptosis. Experimental studies reveal that HeLa cells with knocked-out NCOA4 exhibit weakened ferritinophagy, elevated ferritin levels, increased expression of antioxidant stress genes, and suppressed erastin-induced ferroptosis. Conversely, HeLa cells overexpressing the NCOA4 gene demonstrate contrasting results (Gryzik et al., 2021). In summary, NCOA4-mediated ferritinophagy modulates cytoplasmic Fe2+ levels through multiple pathways, thereby exerting an impact on subsequent lipid peroxidation.

## 4 Regulators affecting ferroptosis and ferritinophagy processes

The research concerning drugs related to ferroptosis and ferritinophagy is gaining momentum. Through the exploration of the mechanisms underlying ferroptosis and ferritinophagy, an array of biomolecules and compounds that influence these processes continue to be discovered and developed (Table 2). Investigating the regulation of ferroptosis has emerged as a potential therapeutic strategy for various diseases. In the realm of cancer, targeting the induction of iron-dependent cell death holds promise for treatment (Mou et al., 2019). In neurodegenerative disorders, cardiovascular diseases, renal disorders, chronic ailments like osteoporosis and osteoarthritis, inhibiting cellular ferroptosis proves beneficial in slowing down disease progression (Qiu et al., 2020; Lin et al., 2022; Miao et al., 2022).

**TABLE 2:** Regulators affecting ferroptosis and ferritophage processes.

Category	Mechanism of action	Related drugs and small molecules
**Ferroptosis inducer**	Inhibit system Xc-	Erastin; Sulfasalazine; Sorafenib; Glutamate
	Inhibit GSH/GPX4 axis	RSL3; Statins; FIN56; FINO2; ML 162; ML 210; Withaferin; BSO; acetaminophen
	Promote iron ion accumulation	Erastin; FINO2; Artemisinin; RSL5
	promote lipid peroxidation	Erastin; Sorafenib
**Ferroptosis inhibitor**	Promote GSH/GPX4 axis	NAC; Fer-1; Lip-1; DFO
	Inhibit lipid peroxidation	Lip-1; α-tocopherol; Panthenol; FSP1; GCH1
	Chelated iron ions	DFO
**Ferritinophagy inducer**	Promotes NCOA4 production	ZnONPs; Arsenite; Sorafenib; IR
**Ferritinophagy inhibitor**	Inhibit NCOA4 expression	3-MA; MT; Compound 9a

System Xc-: An amino acid antiporter located on the cell membrane. GPX4: An enzyme that reduces lipid peroxides to lipid alcohols and thereby protects cell membranes from oxidative damage. NCOA4: A selective cargo receptor mediating ferritinophagy. RSL3: RAS-selective lethal 3; RSL5: RAS-selective lethal 5; FIN56: Ferroptosis inducing 56; BSO: buthionine sulfoximine; NAC: N-acetylcysteine; Fer-1: Ferrostatin-1; Lip-1: Liproxstatin-1; DFO: deferoxamine; FSP1: Ferroptosis suppressor protein 1; GCH1: GTP, cyclohydrolase-1; IR: ionizing radiation; 3-MA: 3-Methyladenine; MT: melatonin.

### 4.1 Ferroptosis promoter and inducer

Predicated upon the intricate mechanisms of ferroptosis, the operational modalities of ferroptosis inducers can be broadly classified into several categories: The initial category functions by thwarting system Xc-to impede the influx of cystine, accomplished primarily by quelling the activity of SLC7A11, thereby orchestrating a decrement in intracellular GSH levels (Feng and Stockwell, 2018). The subsequent category attenuates the resilience of antioxidant defenses by targeting the pivotal GSH/GPX4 axis, encompassing the suppression of GPX4 enzymatic activity, amplification of GPX4’s rate of degradation, and acceleration of GSH depletion. The third stratagem entails fostering an enhanced iron metabolism that ultimately yields an accumulation of iron ions, triggering the cellular cascade of ferroptosis. In the fourth cohort, the deliberate propulsion of lipid peroxidation precipitates the unfolding of cellular ferroptosis.

#### 4.1.1 Inhibiting system Xc - inducing ferroptosis

Erastin emerges as a prominent contender among the extensively studied inducers of ferroptosis. Unearthed in 2003 as a genotype-selective antitumor agent, it exhibits heightened selectivity toward tumor cells harboring RAS mutations, exhibiting pronounced specificity against and enduringly halting the function of system Xc- (Dolma et al., 2003). The cellular demise triggered by erastin, characterized by its absence of classical apoptotic features and insusceptibility to apoptosis inhibitors, has earned the nomenclature “ferroptosis” by Dixon et al. (Dixon et al., 2012). Erastin exerts a specific inhibition on SLC7A11 while sparing SLC3A2 from significant perturbations. This intricate interaction culminates in the targeted repression of cystine uptake and glutamate release facilitated by system Xc-, thereby curtailing the cellular competence for cystine intake and glutamate efflux (Dixon et al., 2014). Concurrently, the attenuation of cystine uptake, a pivotal precursor for GSH synthesis, triggers a concomitant reduction in GSH production. Consequently, the efficacy of GPX4, the enzyme reliant on GSH for peroxide degradation, wanes due to the diminished availability of GSH (Shiromizu et al., 2019). Gout demonstrated that 0.3 mM sulfasalazine specifically inhibits system Xc-, significantly suppressing the growth of rat lymphoma, highlighting its effectiveness as a system Xc-inhibitor, which curtails cystine absorption and leads to GSH reduction (Gout et al., 2001). On the other hand, sorafenib can indirectly downregulate the expression of SLC7A11 in hepatic stellate cells by reducing the expression of hypoxia-inducible factor 1-alpha (HIF-1α), thereby depleting GPX4 and GSH and resulting in iron, ROS, and MDA accumulation (Yuan et al., 2022). Sorafenib induces ferroptosis in hepatic stellate cells through the HIF-1α/SLC7A11 signaling pathway, thereby mitigating liver injury and fibrosis. The inhibition of system Xc-has the most profound impact on the depletion of cysteine, a precursor of glutathione synthesis. As a transporter for the reverse transport of glutamine and cysteine, system Xc-is influenced by the concentration of glutamate, which further affects cellular cysteine intake (Dixon et al., 2014).

#### 4.1.2 Inhibiting GSH/GPX4 axis inducing ferroptosis

There is a category of drugs that induce ferroptosis by inhibiting the GSH/GPX4 axis, including RAS-Selective Lethal 3 (RSL3), statins, Ferroptosis Inducing 56 (FIN56), FINO2, ML 162, ML 210, and Withaferin. These drugs act by suppressing the activity of GPX4 or degrading GPX4, preventing the conversion of lipid hydroperoxides into corresponding lipid alcohols. This disruption leads to iron-dependent toxic lipid peroxidation, generating ROS and promoting lipid peroxidation. Yang et al. identified a ferroptosis inducer called RSL3, which stimulated lipid ROS production in BJeLR cells without altering GSH levels (Yang and Stockwell, 2008). Several studies have pinpointed GPX4 as the target of RSL3, with selenocysteine serving as the nucleophilic amino acid residue at the active site of GPX4. RSL3 can interact with selenocysteine and GPX4 (Yang and Stockwell, 2008; Yang et al., 2014). Treatment of HT-1080 cells with fluvastatin resulted in delayed and dose-dependent reduction in GPX4 expression, subsequently diminishing cellular resistance against lipid peroxidation (Viswanathan et al., 2017). FIN56, a CIL56 analog, led to GPX4 protein degradation when applied to BJeLR cells, ultimately inducing ferroptosis (Shimada and Stockwell, 2016). Through organic peroxide screening, FINO2 was identified as a compound containing a 1,2-dioxolane ring, indirectly inhibiting GPX4 enzyme activity and promoting ferroptosis (Abrams et al., 2016; Gaschler et al., 2018). Unlike previously described ferroptosis inducers, FINO2 does not inhibit system Xc- or directly target the reductase enzyme GPX4, nor does it deplete GPX4 protein as FIN56 does. Instead, FINO2 exerts its effect by both indirectly inhibiting GPX4 enzymatic function and directly oxidizing iron, ultimately leading to widespread lipid peroxidation. HRAS, a mutated oncogenic RAS gene, was targeted by ML162, causing selective lethality in HRAS-expressing BJeLR cells (Figueiredo et al., 2012). It has now been discovered that ML162 may not act as a direct biochemical inhibitor of GPX4, adding a layer of complexity to our understanding (Cheff et al., 2023). Withaferin, a natural plant-derived compound from the nightshade family, was found to enhance lipid peroxidation in IMR-32 neuroblastoma cells. It could also bind to Cys107 in GPX4, leading to the loss of GPX4 enzyme activity (Hassannia et al., 2018).

Two compounds, Buthionine sulfoximine (BSO) and acetaminophen, can expedite the depletion of GSH, diminish cellular antioxidant capacity, and propel lipid peroxidation. At the epicenter of GSH synthesis stands glutamate-cysteine ligase, a pivotal enzyme governed by the throttling influence of BSO. By virtue of this influence, GSH production encounters inhibition, ushering in a cascade of cellular responses (Yang et al., 2014). Acetaminophen, a commonplace therapeutic agent, embarks on a journey of hepatic metabolism. Predominantly, it finds its exit route through conjugation with sulfate or glucuronide entities, facilitating its excretion from the body’s precincts. However, a lesser fraction of acetaminophen embarks on a unique metabolic pathway involving cytochrome P450 enzymes, culminating in the formation of N-acetyl-p-benzoquinone imine (NAPQI). The remarkable propensity of NAPQI lies in its swift interaction with GSH, instigating a rapid depletion of this critical cellular guardian (Lőrincz et al., 2015). The ramifications of this phenomenon were explored in a study where the ramifications of excessive acetaminophen exposure upon C57BL/6 mice and LO2 cell lines were scrutinized. A study exposing C57BL/6 mice and LO2 cell lines to excessive acetaminophen discerned an elevation in iron content, ROS, MDA, and 4-HNE.Equally noteworthy, the strategic intervention of the ferroptosis inhibitor ferrostatin-1 offered a palpable amelioration, effectively tempering the cellular toxicity attributed to acetaminophen’s influence (Wang C. et al., 2021).

#### 4.1.3 Iron accumulation inducing ferroptosis

Erastin can elevate TFR levels through ROS-induced autophagy and facilitate the degradation of FTH1, thus orchestrating the regulation of intracellular iron levels. This orchestrated modulation culminates in the induction of iron-dependent ferroptosis (Park and Chung, 2019). In a distinctive mechanism, FINO2 directly oxidizes ferrous ions, fostering an accumulation of iron ions that precipitates extensive lipid peroxidation. This sequence of events eventually ushers in iron-dependent cell death (Abrams et al., 2016; Gaschler et al., 2018). A notable category of antimalarial drugs, including artemisinin and its derivatives, has garnered recent attention due to their intricate interplay with iron-associated gene expression. The expression of mRNA encoding for TF, TFR1, TFR2, and other such genes, exhibits a significant correlation with artemisinin concentration. Remarkably, the presence of ferroptosis inhibitor ferrostatin-1 and iron-chelating agent deferiprone appears to alleviate the cellular toxicity imposed by artemisinin and its derivatives (Ooko et al., 2015).

#### 4.1.4 Promotes lipid peroxidation to induce ferroptosis

Lipid peroxidation is central to ferroptosis, facilitated by iron ions catalyzing ROS, leading to the loss of cell membrane integrity and cell death. Maintaining a balance of iron ions and lipid peroxidation is crucial in preventing ferroptosis. Erastin also exerts its influence by modulating the activity of ACSL4, thereby fine-tuning the landscape of lipid peroxidation. This modulation stems from ACSL4’s pivotal role in promoting the esterification of arachidonic acid (AA) and adrenic acid (AdA) into phospholipids, resulting in the formation of AA/AdA-PE derivatives that are more prone to oxidative events (Yuan et al., 2016). Recognized the inherent metabolic instability of erastin within the biological milieu, attention has shifted towards its derivatives, such as piperazine-erastin and imidazolone-erastin, which exhibit enhanced metabolic stability within the body. These derivatives hold considerable promise as robust contenders for inducing iron-dependent cell death (Zhang Y. et al., 2019). In a similar vein, sorafenib emerges as a noteworthy contributor to this intricate landscape. Its action revolves around the meticulous modulation of ACSL4 enzyme activity, engendering an amplified pool of AA/AdA-PE molecules, ultimately fueling the propagation of lipid peroxidation (Lei et al., 2020; Ye et al., 2020).

### 4.2 Ferroptosis inhibitor

There are primarily three mechanisms for inhibiting iron-dependent cell death. Firstly, suppression of iron-dependent cell death is achieved by upregulating the activity of the GSH/GPX4 axis. Compounds such as N-acetylcysteine (NAC) can elevate GSH levels, while Ferrostatin-1 (Fer-1), Liproxstatin-1 (Lip-1), and deferoxamine (DFO) enhance GPX4 enzyme activity. DFO additionally enhances the ability of system Xc-to transport cystine, further dampening iron-dependent cell death. The second mechanism involves the inhibition of lipid peroxidation, a crucial process underlying iron-dependent cell death. Noteworthy compounds within this category encompass Lip-1, tocopherol, coenzyme Q10 (CoQ10), ferroptosis suppressor protein 1 (FSP1), and tetrahydrobiopterin (BH4) (Stockwell and Jiang, 2020). The third avenue revolves around the chelation of iron ions, thereby decelerating the progression of iron-dependent cell death. Compounds like deferoxamine (DFO) and deferiprone (DFP) stand as exemplars in this category. These compounds manifest the capacity to reverse cellular iron-dependent cell death and, in doing so, may emerge as promising therapeutic agents.

#### 4.2.1 Upregulate the GSH/GPX4 axis to inhibit ferroptosis

NAC functions as a precursor to L-cysteine and the reduced form of GSH. Notably, NAC promotes GSH synthesis, fortifying cellular defenses against oxidative stress. Beyond this, NAC’s direct scavenging interaction with free radicals confers its pronounced antioxidant properties, coupled with a capability to remediate damaged molecular targets. As a cysteine donor in reduced GSH biosynthesis, NAC amplifies the intracellular antioxidative machinery, curtails lipid peroxidation, and mitigates ferroptotic pathways (Tardiolo et al., 2018). Following glutamate exposure in HT22 cells, an augmentation in mitochondrial membrane density is observed alongside a diminution in GPX4 enzymatic vigor. Contrastingly, a 12-h Fer-1 pre-incubation restores GPX4 activity and attenuates ROS generation, underscoring Fer-1’s potential in shielding cells from glutamate-elicited oxidative lethality (Chu et al., 2020; Miotto et al., 2020). High-throughput screening of a small-molecule library unveiled Lip-1, a stalwart entity in the domain. Subsequent experimentation in an ischemia/reperfusion model of murine hearts demonstrated that following a 2-h exposure to 200 nM Lip-1, the degradation of voltage-dependent anion channel 1 (VDAC1) within the myocardium escalated. Moreover, Lip-1 exhibited a gift for elevating GPX4 levels and attenuating mitochondrial Complex I-mediated ROS generation (Feng et al., 2019). Within primary cortical neuronal cells, the ROS surge induced by erastin finds mitigation when exposed to 50 μM DFO for a span of 12 h, showcasing its efficacy in ROS mitigation compared to the control group (Zhang et al., 2020). DFO effectively mitigates ROS levels and thwarts erastin-induced cell death by modulating the GPX4/xCT signaling pathway.

#### 4.2.2 Interruption of lipid peroxidation inhibits ferroptosis

Lip-1 plays a pivotal role in modulating ferroptotic processes by effectively neutralizing lipid peroxides and free radicals (Sheng et al., 2017). Mechanistically, Lip-1 counters ferroptosis in the C57BL/6J murine model through the attenuation of 15-lipoxygenase (15-LOX) enzymatic action (Sheng et al., 2017; Feng et al., 2019). α-Tocopherol, commonly referred to as vitamin E, emerges as a formidable scavenger of free radicals, endowed with the capability to thwart phospholipid hydroperoxide genesis. Significantly, the inherent affinity of α-tocopherol for unpaired electrons translates into its ability to disrupt the cascade of events in membrane lipid peroxidation, thus providing a bulwark against cellular ferroptosis (Angeli et al., 2017). Coenzyme Q10 (CoQ10), an intrinsic vitamin within the human milieu, finds its reduced counterpart in ubiquinol. Ubiquinol, pervasively dispersed within biological membranes and low-density lipoproteins, confers antioxidative protection, thereby safeguarding cells from oxidative duress (Takayanagi et al., 1980; Bersuker et al., 2019). Intriguingly, FSP1-ablated cells treated with RSL3 exhibited heightened susceptibility to ferroptosis. This unique phenotype could be ameliorated by ferroptosis inhibitors but remained impervious to interventions involving apoptosis and necroptosis inhibitors. This underscores the potential of FSP1 as a strategic ferroptosis modulator (Gotorbe et al., 2022), with the noteworthy distinction that its inhibitory prowess is GSH-independent (Doll et al., 2019). The FSP1-CoQ10-NAD(P)H pathway operates as an independent and parallel system, collaboratively working with GPX4 and glutathione to inhibit phospholipid peroxidation and ferroptosis. Tetrahydrobiopterin (BH4) functions as a pivotal cofactor in neurotransmitter biosynthesis and a plethora of chemical transformations. BH4, serving as an efficacious free radical scavenging antioxidant, safeguards lipid membranes from spontaneous oxidation. The upregulation of GTP cyclohydrolase-1 (GCH1) catalyzes the synthesis of the BH4/BH2 tandem, constituting an effective countermeasure against lipid peroxidation (Kraft et al., 2020; Soula et al., 2020). This protective role underscores its significance in maintaining cellular integrity and defending against oxidative damage.

#### 4.2.3 Chelate iron ions to inhibit ferroptosis

Given that iron accumulation stands as the principal pathological event of ferroptosis, iron chelators such as DFO and DFP, capable of lowering intracellular iron content, emerge as strategic inhibitors of the genesis and progression of ferroptosis (Dixon et al., 2012). Iron chelators agent promote iron excretion and reduce iron accumulation in the body. DFO, beyond its capacity to elevate system Xc- and GPX4, extends its mastery by chelating iron ions, curbing the onset of ferroptosis. In the theater of *in vitro* experiments, where pancreatic islet cells faced the crucible of erastin and RSL3 treatment for 24 h, a stark proliferation of cell death prevailed. Yet, this bane was allayed when pancreatic islet cells were pre-treated with DFO (Bruni et al., 2018). Intraperitoneal injection of DFO can effectively reduce iron levels in mice, and can significantly save the deterioration of bone microarchitecture exposed to irradiation (Zhang et al., 2019b). DFO also reduces the amount of iron in the bones, reducing bone loss due to hindlimb unloading (Xu et al., 2017). DFO binds virtually all internal iron, precluding the orchestration of further ROS generation (Ibrahim et al., 2006; Liu et al., 2018). Deferiprone, a familiar constituent in the therapeutic arsenal against severe cases of β-thalassemia with iron overload, extends its reach beyond, facilitating the transport of bound iron to both intra- and extra-cellular iron receptors (Sripetchwandee et al., 2016). While DFO has achieved certain therapeutic effects in various iron-overload disease mouse models, its future clinical application faces challenges due to several factors. The compound exhibits numerous side effects and poor liposolubility, and it is constrained by a singular mode of administration. These limitations necessitate meticulous consideration and potentially further refinement for optimized therapeutic utilization in the future.

### 4.3 Ferritinophagy regulator

The role of iron in lipid peroxide generation and the initiation of ferroptosis remains pivotal. Sensitivity to ferroptosis is intricately entwined with the availability of the LIP and the dynamic interplay of proteins that orchestrate intracellular iron homeostasis. A bidirectional regulatory mechanism, orchestrated by NCOA4-mediated Ferritinophagy, governs the content of Fe2+ within the cellular LIP. Within physiologic confines, Ferritinophagy maintains iron balance. In scenarios where cellular iron becomes inadequate, NCOA4-mediated Ferritinophagy augments the Fe2+ content within the LIP. Conversely, surplus intracellular iron prompts HERC2 to orchestrate NCOA4’s ubiquitin-dependent degradation, thus curbing the Fe2+ content in the LIP (Mancias et al., 2015). Suppression of Ferritinophagy finds its expression in the blockade of autophagy or the attenuation of NCOA4 expression, thwarting the buildup of labile iron and reactive oxygen species integral to ferroptosis (Gao et al., 2016). Studies by Yang et al. illuminate that silencing NCOA4 within SD rat AF and NP cells culminates in elevated GPX4 content, accompanied by a decline in lipid peroxidation levels. Notably, autophagic markers, such as P62 and LC3, manifest negligible alterations, signifying the potential for NCOA4 silencing to mitigate ferroptosis, unaffected by changes in Ferritinophagy activity (Yang RZ. et al., 2021). As NCOA4 directly regulates ferritin levels, thereby modulating the bioavailability of iron within cells, NCOA4-mediated Ferritinophagy emerges as a regulator of cellular ferroptosis (Liu et al., 2020). In a study, zinc oxide nanoparticles (ZnONPs) induced elevated iron levels and lipid peroxidation in HUVEC and EA. hy926 cells, ultimately triggering ferroptosis. Furthermore, ZnONPs-induced ferroptosis was dependent on NCOA4-mediated degradation of FTH (Qin X. et al., 2021). Arsenic trioxide, a neurotoxic metal salt, induced dose-dependent ferroptosis in neuronal cells. Notably, arsenic trioxide led to a significant reduction in ferritin and NCOA4 levels, while the autophagic marker LC3B showed marked elevation. The use of Ferritinophagy inhibitor 3-methyladenine (3-MA) attenuated the cytotoxic effects of arsenic trioxide (Xiao et al., 2021). Additionally, Zhou et al. found that downregulation of NCOA4 inhibited ionizing radiation (IR)-induced ferroptosis in HIEC cells (Zhou H. et al., 2022). This suggests that IR may disrupt the intracellular iron balance through NCOA4, thereby inflicting damage on cells. Furthermore, sorafenib promoted Ferritinophagy by increasing NCOA4 expression in hepatocellular carcinoma cells (Louandre et al., 2015).

Among the plethora of autophagy inhibitors, 3-MA is one of the most extensively employed agents. The seminal work of Seglen and others in 1982 revealed that 3-MA exerts its inhibitory influence on the degradation of autolysosomal proteins within rat liver cells (Seglen and Gordon, 1982). 3-MA primarily works by inhibiting the activity of Phosphoinositide 3-Kinase, thereby blocking the initial stages of autophagy. PI3K is a key enzyme involved in the formation of autophagic membranes, hence, the addition of 3-MA results in a reduction of autophagosomes, inhibiting the autophagy process. Delving into the intricacies of cellular responses, Melatonin (MT) can suppress the expression of NCOA4, thereby inhibiting UVB-induced ferroptosis in B-3 cells (Mi et al., 2023). Recent scientific explorations unveiled an innovative harbinger of cell survival, 9a, a newly discovered ferroptosis inhibitor. With a precision akin to a maestro, 9a orchestrates its impact by selectively binding to NCOA4. This interaction disrupts the liaison between NCOA4 and FTH1, culminating in a cascade of events that culminate in the reduction of the cellular LIP and a definitive cessation of the ferroptosis narrative (Fang et al., 2021).

## 5 The roles of ferroptosis and ferritinophagy in arthritis

Numerous studies have substantiated the involvement of ferroptosis and ferritinophagy in the onset and progression of musculoskeletal disorders. Upstream factors intricately orchestrate the progression of musculoskeletal disorders through the regulation of ferroptosis and ferritinophagy. A plethora of investigations have illuminated the characteristic alterations of ferroptosis and ferritinophagy, including system Xc-dysfunction, aberrant iron metabolism, and lipid peroxidation, across diverse musculoskeletal conditions such as osteoarthritis, osteosarcoma, and osteoporosis. These insights underscore the potential of targeting ferroptosis and ferritinophagy as novel therapeutic strategies for musculoskeletal disorders.

### 5.1 Osteoarthritis

Osteoarthritis (OA) constitutes a progressive degenerative ailment that stands as a prominent contributor to adult disability. Its etiology is multifaceted, influenced by genetic predisposition, advancing age, traumatic events, and intricate biomechanical interplays. Characterized by a complex interplay of pathological manifestations within the entire synovial joint, OA is typified by the degeneration of cartilage, inflammatory responses within the synovial lining, subchondral bone deterioration, and hypertrophic tissue changes (Yao et al., 2023). Epidemiological surveys conducted in 2017 underscore a global prevalence of OA at 3754.2 cases per 100,000 individuals, with an annual incidence of 181.2, showcasing a respective increase of 9.3% and 8.2% since 1990. These statistics underscore the mounting significance of OA as a substantial public health dilemma (Safiri et al., 2020). Existing therapeutic approaches for OA predominantly concentrate on mitigating clinical pain symptoms, optimizing joint function, and enhancing bone density. However, these interventions often fall short of thwarting the relentless progression of the disease. In recent times, the focus of OA management has transitioned towards preemptive measures, aiming to defer the advancement of OA prior to widespread cartilage damage (Yao et al., 2023). Recent research endeavors have unveiled a compelling nexus between ferroptosis phenomena in chondrocytes and the trajectory of OA progression.

Inflammation stands as a crucial driving force underlying the progression of OA. Chondrocytes exposed to IL-1β manifest a notable surge in ROS, triggering the activation of matrix metalloproteinases (MMPs), including MMP-13, MMP-3, and MMP-9, which participate in the breakdown of the extracellular matrix (Khan et al., 2017), and the number of osteoclasts increased (Jing et al., 2020). Furthermore, the influence of IL-1β extends to inhibiting chondrocyte proliferation and promoting apoptosis. This is discerned through the amplified release of apoptosis-associated proteins like caspase-3 and BAX, accompanied by a decrease in the levels of the anti-apoptotic protein BCL-2 (Zhou et al., 2019). Previous investigations reported the substantial elevation of iron content within the joint fluid of individuals affected by OA (Yazar et al., 2005), with serum ferritin levels in OA patients correlating positively with the severity of cartilage damage in the knee joint (Kennish et al., 2014). Employing a murine model of iron overload established with dextran iron, iron-overloaded mice display accentuated cartilage degradation and subchondral bone loss (Jing et al., 2020). Within chondrocytes experiencing iron overload, there emerges an escalation in the expression of TFR1 and DMT1, while FPN expression dwindles (Jing et al., 2020). Furthermore, the generation of hydroxyl radicals via the Fenton reaction contributes to a dip in mitochondrial membrane potential, underscoring a state of mitochondrial dysfunction (He et al., 2023). Mitochondria, known for their dynamic nature, emerge as central players in the context of OA pathogenesis. The age-dependent decline of mitochondrial deacetylase SIRT3 results in augmented post-translational acetylation, leading to a reduction in the activity of superoxide dismutase 2 (SOD2) (Fu et al., 2016). Diminished SOD2 activity not only curtails the expression of enzymes responsible for matrix degradation but also heightens lipid peroxidation and mitochondrial damage within chondrocytes (Gavriilidis et al., 2013). While the immediate mitigation of hydrogen peroxide (H2O2) production rates can serve as a defense against oxidative stress, a transition takes place over time, shifting towards fostering cellular oxidative damage (Dasgupta et al., 2006).

It is widely recognized that excessive mechanical loading culminates in evident cartilage degeneration. Anomalies in mechanical loading notably suppress the expression of Sod2, which in turn disrupts extracellular matrix metabolism through an increase in mitochondrial superoxide accumulation (Koike et al., 2015). In patients with OA who have undergone total knee replacement, clinical cartilage samples conspicuously reveal cartilage damage in the loading zone—where mechanical pressure is concentrated—when compared to the relatively dispersed unloading zone. Detailed observation through transmission electron microscopy (TEM) uncovers ferroptosis characteristics in chondrocytes within the loading zone, characterized by mitochondrial membrane thickening and mitochondrial contraction. Concurrently, the RNA levels of GPX4, a pivotal inhibitor of iron-mediated cell death, witness a decline under the influence of mechanical loading. This pattern is also confirmed through *in vitro* experiments employing TEM, revealing analogous reductions in GPX4 protein and mRNA levels. Delved further, investigations highlight that as mechanical loading escalates, calcium ions permeate chondrocytes in tandem with the activation of the Piezo1 channel. This occurrence subsequently leads to reduced levels of GSH, heightened production of ROS, a further decrease in GPX4 expression, and a compromised mitochondrial membrane potential. The cumulative effect amplifies the propensity for iron-mediated cell death and mitochondrial dysfunction in chondrocytes (Wang et al., 2022). Moreover, repetitive mechanical loading leads to the accumulation of the protein gremlin-1 in the deep layers of articular cartilage, amplifying the activation of NF-κB (Chang et al., 2019), thus prompting chondrocyte apoptosis and degradation (Ulivi et al., 2008), osteochondral ossification (Caron et al., 2012), contributing to disturbances in the homeostasis of articular cartilage.

Recent investigations have illuminated the influence of NCOA4-mediated ferritinophagy on the progression of OA. Enhanced NCOA4 expression has been noted in the articular cartilage of individuals afflicted with OA, as well as in murine models of post-traumatic OA. Notably, there exists a direct positive correlation between the expression of NCOA4 and the advancement of OA. When the NCOA4 gene is selectively silenced within chondrocytes, intriguing shifts occur. In chondrocytes where the NCOA4 gene is knocked down, degradation of FTH1 and a notable reduction in Fe2+ levels were observed. Moreover, heightened levels of GSH are discerned, concomitant with a reduction in levels of representative ROS and MDA, hallmark indicators of lipid peroxidation. Furthermore, insights gleaned from TEM reveal the intriguing reversal of mitochondrial shrinkage and the restoration of mitochondrial cristae, a notable observation that suggests the potential for the reversal of NCOA4 gene silencing to counteract the ferroptosis pathway elicited by IL-1β (Sun et al., 2023). Recent studies reveal connections between ferroptosis in chondrocytes and OA progression, with iron overload and mitochondrial dysfunction playing significant roles. Inflammation, characterized by increased ROS and matrix degradation, and mechanical loading, resulting in mitochondrial damage and suppressed antioxidant expression, are key contributors. Additionally, NCOA4-mediated ferritinophagy has been identified as influential in OA progression, with changes in this pathway impacting iron levels, oxidative stress, and mitochondrial structure, offering potential therapeutic avenues.

### 5.2 Rheumatoid arthritis

Rheumatoid arthritis (RA) is an autoimmune disorder characterized by its capacity to invade multiple joints, including the knees and elbows. RA affects approximately 0.5⁓1% of the global population, with a peak incidence between the ages of 50 and 59. Notably, the female prevalence is 2⁓3 times higher than in males. However, the predominant therapeutic approach currently focuses on maximal mitigation of symptoms and enhancement of physical function, all while avoiding the potential generation of subsequent sequelae (Smolen et al., 2016; Smith and Berman, 2022). The chief pathological changes in RA involve synovitis and the formation of vascular pannus, leading to progressive destruction of joint cartilage and bone tissue (Guo et al., 2020). Patients commonly manifest early morning joint swelling and stiffness, a harbinger of the impending trajectory towards joint deformities and functional compromise in the absence of timely intervention (Huang et al., 2021).

In 1968, research findings had already illuminated a substantial elevation in iron content within the synovial tissue of individuals grappling with RA (Senator and Muirden, 1968) More recently, scientific inquiries have unveiled a nuanced interplay involving the altered expression of iron-binding protein ferritin and diminished saturation of transferrin within the blood of RA patients (Tański et al., 2021). In consequence, discernibly diminished iron levels stand in contrast to those found in the control group. Despite experiencing a partial recuperation in patients in a state of RA remission, the levels of iron fail to reach the benchmarks witnessed in the control group. Moreover, a negative correlation emerges between circulating iron and the inflammatory marker CRP, implying an inverse relationship between iron levels and disease activity (Wang et al., 2023). This intriguing observation is speculated to be a result of the overexpression of hepcidin, a regulator that prompts the localized deposition of iron, subsequently triggering a reduction in peripheral blood iron content (Sato et al., 2020). Importantly, the introduction of iron-dextran via joint injections further exacerbates synovitis by catalyzing lipid peroxidation and depleting intracellular GSH levels within red blood cells (Blake et al., 1985; Roberts and Davies, 1987). Within a human synovial cell model simulating synovitis via lipopolysaccharide (LPS) induction, conspicuous increases in iron and MDA levels become apparent. Among proteins implicated in iron-induced cell death, TFR1 and NCOA4 manifest prominent upregulation as contributors to this phenomenon. Conversely, the expression levels of antioxidant proteins, including GPX4, SLC7A11, and SLC3A2, are noticeably reduced, indicating a modulated state (Luo and Zhang, 2021). This unequivocally highlights the participation of iron-induced cell death and ferritinophagy in propelling the progression of synovitis. Additionally, investigations have illuminated escalated levels of ROS within tissues affected by RA as compared to their healthy counterparts (Winyard et al., 2011). The conspicuous synovial hyperplasia that characterizes rheumatoid arthritis infiltrates cartilage and bones, culminating in progressive joint degeneration. Over the course of RA, activated macrophages release a multitude of cytokines, including TNF-α, IL-1β, IL-6, and B-cell activating factor (BAFF). The activation of TNF-α initiates Bid, a BH3 domain member within the Bcl2 family, thereby inducing ROS generation at the cell surface through NADPH oxidase (Latchoumycandane et al., 2012). Importantly, synovitis emerges as a hallmark pathology of RA, with fibroblast-like synoviocytes (FLSs) constituting a predominant cell type within the proliferative tissue. Stimulated by TNF, FLSs generate a spectrum of inflammatory cytokines (Du et al., 2019), coupled with matrix-degrading MMP3, MMP9, and MMP13 (Wang XH. et al., 2021). This convergence results in the inhibition of cartilage proteoglycan synthesis and the provocation of chondrocyte apoptosis, furthering joint deterioration. The activation of the NF-κB pathway by TNF enhances cysteine uptake and GSH generation, effectively buffering FLSs against oxidative stress and the impact of excessive iron. Consequently, FLSs can proliferate abnormally despite heightened ROS and lipid peroxidation (Wu et al., 2022). Furthermore, ROS fuels the production of cytokines such as TNF-α, IL-1β, and IL-6 (Mirshafiey and Mohsenzadegan, 2008; Mateen et al., 2017). Elevated inflammatory mediators precipitate lipid anomalies during the course of RA progression, culminating in low-density lipoprotein oxidation. This culminates in the exacerbation of inflammation through the upregulation of chemokines, adhesion molecules, and advanced glycation end products (Phull et al., 2018), resulting in a cyclic interplay between inflammation and ROS. Notably, ROS also orchestrates FLS migration and contributes to the formation of vascular pannus, thus accelerating the onset and advancement of RA (Zhou et al., 2012). The disease sees an intricate relationship between elevated iron levels in synovial tissue, inflammation, and progression of synovitis, attributed to the overexpression of hepcidin and the role of iron-induced cell death. Increased ROS levels contribute to joint degeneration, inflammation, and abnormal proliferation of FLS. The interaction of cytokines and the NF-κB pathway exacerbates inflammation and accelerates RA progression, highlighting a complex interplay between iron metabolism, ROS, and inflammatory responses in RA.

### 5.3 Gouty arthritis

Gout ranks among the most prevalent forms of global inflammatory arthritis. It is distinguished by the accrual of monosodium urate (MSU) crystals within joints and the encompassing periarticular milieu. This pathogenic cascade is spurred by the elevation of serum uric acid (SUA) levels, culminating in the transgression of physiological saturation thresholds governing uric acid concentrations within bodily fluids (Rees et al., 2014). Foremost clinical presentations of gout encompass intense pain in peripheral joints, notably the first metatarsophalangeal joint, alongside joint impairment and the deposition of MSU crystals in the skin. Driven by shifts in lifestyle, dietary patterns, and an aging demographic, the incidence of gout exhibits an upward trajectory (Roddy and Doherty, 2010).

Achieving serum uric acid reduction below the point of urate saturation represents an effective “cure” for gout. A nationwide population study conducted in China demonstrated that levels of serum ferritin (SF), transferrin, and soluble transferrin receptor (sTfR) are positively associated with the risk of hyperuricemia (Li et al., 2018). Similar findings have emerged from population studies conducted in different geographical areas. Investigations involving New Zealand Polynesian and United States populations highlighted a direct correlation between elevated ferritin levels and the frequency of gout attacks (Fatima et al., 2018). Additionally, studies utilizing serum iron, ferritin, transferrin saturation, and transferrin levels have revealed a positive relationship between hereditary high iron status and gout incidence (Yuan and Larsson, 2020). In a cross-sectional study, subjects with hyperuricemia were found to have significantly elevated serum ferritin levels, and their levels were positively associated with the prevalence of hyperuricemia (Zhang et al., 2014). An intriguing clinical trial revealed that maintaining iron levels in hyperuricemia patients with gouty arthritis near a state of near-iron deficiency (NID) led to significant reductions in the frequency of gout attacks over a 28-month NID maintenance period, often accompanied by reduced attack severity (Facchini, 2003). In a study by Du et al., it was observed that joint tissues of mice with gouty arthritis exhibited marked increases in free iron, NO, and MDA levels, while GSH levels were notably diminished (Du et al., 2023). Research across various regions has identified a consistent positive correlation between elevated serum ferritin, transferrin, and iron levels and the risk of hyperuricemia and gout attacks. Maintaining near-iron deficiency has been proven to significantly reduce gout attack frequency and severity. Furthermore, animal studies, particularly in mice, have shown that gouty arthritis is associated with increased levels of free iron, NO, and MDA, and decreased GSH levels in joint tissues.

## 6 Conclusion and prospect

Iron, a vital micronutrient in the human body, assumes a pivotal role in regulating essential metabolic functions. However, the accumulation of iron can perturb the delicate balance between oxidative and antioxidant stress systems, provoking an excessive generation of reactive oxygen species and lipid peroxidation. These disruptions are implicated in the occurrence of iron-induced cell death. In recent times, there has been a surge in research concerning the disturbances in intracellular iron equilibrium brought about by various diseases. In the course of the development of numerous joint ailments, significant shifts have been observed in the expression levels of proteins linked to iron metabolism and enzymatic activities. Thus, efforts to alleviate and rectify these imbalances in iron homeostasis have progressively become focal areas of investigation.

Ferroptosis, a recently discovered crucial form of regulated cell demise, emerges as a consequence of cellular iron overload that triggers lipid peroxidation. Ferritinophagy can promote the progression of ferroptosis by affecting the degradation of ferritin to increase the amount of iron in the cell. Research focus on ferroptosis has progressively extended beyond the realms of oncology, encompassing diverse fields of scientific exploration. Within the purview of orthopedics, where joint disorders are rife, ferroptosis appears to be assuming an increasingly pivotal role. It is worth noting that the complexity of orthopedic diseases, involving distinct cellular constituents such as osteoblasts, osteoclasts, chondrocytes, and synovial cells, engenders diverse ramifications of ferroptosis during the progression of various pathologies. As a corollary, tailored interventions for ferroptosis manifest distinct paradigms, encompassing mechanisms like the modulation of the GSH/GPX4 axis, inhibition of lipid peroxidation, and chelation of iron ions. According to the mechanism of bone formation, influencing ferroptosis and ferritinophagy to improve bone changes in disease, and then altering the course of disease seems to be a bright direction. In the current panorama, the enigma of ferroptosis’s latent mechanisms within the etiology of orthopedic disorders, particularly in the context of joint afflictions, remains partially unveiled. However, with the gradual deepening of research endeavors, a cornucopia of intricate mechanisms and targeted therapeutic strategies are poised to be unearthed. These potential therapeutic strategies may include the development of new drugs to regulate iron homeostasis, intervention in specific pathways of ferroptosis, and research on how to improve the treatment outcomes of joint diseases by regulating the expression of proteins related to iron metabolism. As we gain a deeper understanding of the mechanisms of ferroptosis, we can anticipate the development of more effective treatment methods to address these diseases.
